# GABA_B_ receptor-dependent bidirectional regulation of critical period ocular dominance plasticity in cats

**DOI:** 10.1371/journal.pone.0180162

**Published:** 2017-06-29

**Authors:** Shanshan Cai, Quentin S. Fischer, Yu He, Li Zhang, Hanxiao Liu, Nigel W. Daw, Yupeng Yang

**Affiliations:** 1 CAS Key Laboratory of Brain Function and Diseases, School of Life Sciences, University of Science and Technology of China, Hefei, China; 2 Department of Ophthalmology and Visual Sciences, Yale University School of Medicine, New Haven, Connecticut, United States of America; Nanjing University, CHINA

## Abstract

Gama amino butyric acid (GABA) inhibition plays an important role in the onset and offset of the critical period for ocular dominance (OD) plasticity in the primary visual cortex. Previous studies have focused on the involvement of GABA_A_ receptors, while the potential contribution of GABA_B_ receptors to OD plasticity has been neglected. In this study, the GABA_B_ receptor antagonist SCH50911 or agonist baclofen was infused into the primary visual cortex of cats concurrently with a period of monocular deprivation (MD). Using single-unit recordings we found that the OD shift induced by four days of MD during the critical period was impaired by infusion of the antagonist SCH50911, but enhanced by infusion of the agonist baclofen. In contrast, seven days of MD in adult cats did not induce any significant OD shift, even when combined with the infusion of SCH50911 or baclofen. Together, these findings indicate that an endogenous GABA_B_ receptor-mediated inhibition contributes to juvenile, but not adult, OD plasticity.

## Introduction

Altering visual experience by MD (a model of amblyopia) results in the rewiring of cortical circuitry with visual representation shifting predominantly to the open eye. This phenomenon, OD plasticity, is most robust during a developmental critical period. The excitatory-inhibitory balance appears crucial for OD plasticity. A wealth of evidence indicates that the strength of GABAergic inhibition plays an essential role in the waxing and waning of OD plasticity during development [1, 2]. Knockout of the GABA synthetic enzyme GAD65 prevents the onset of the critical period [3], while reducing intracortical GABAergic inhibition promotes OD plasticity in adults [4–7]. Many of these effects are blocked by diazepam [3, 6], indicating a requirement for GABA_A_ receptor activation, but cortical inhibition may also be mediated by GABA_B_ receptors.

GABA_B_ receptors are metabotropic G protein-coupled receptors [8] located on the soma, dendrites, pre- and post-synaptic sites of inhibitory and excitatory neurons throughout the brain, including the visual cortex [9]. An examination of plasticity at unitary connections between GABAergic fast-spiking cells and pyramidal cells has implicated a GABA_B_ receptor-dependent process in the onset of the critical period [10]. However, the role of GABA_B_ receptors in OD plasticity remains largely unexplored.

*In vitro* synaptic plasticity studies suggest that GABA_B_ receptors may contribute to OD plasticity. GABA_B_ receptors have been shown to regulate the induction of long-term potentiation (LTP) and long-term depression (LTD) at inhibitory and excitatory synapses. In the visual cortex, GABA_B_ receptor activation is necessary for the induction of inhibitory LTP at fast-spiking cell to pyramidal cell synapses, which converts LTP to LTD at convergent excitatory pyramidal cell synapses and is occluded by MD [11, 12]. Additionally, GABA_B_ receptors are crucial for presynaptic cannabinoid receptor-dependent inhibitory LTD, while OD plasticity in layer II/III is impaired by pharmacological blockade of cannabinoid receptors [13–15]. In other brain regions, GABA_B_ receptor agonists enhance, and antagonists inhibit, excitatory LTD [16, 17]. Moreover, GABA_B_ receptor antagonists, or knockout of GABA_B_ receptor B1a subunits, impair excitatory LTP [18, 19]. Since LTD and LTP at inhibitory and excitatory synapses have been hypothesized to be crucial for the attenuation of deprived eye responses and the strengthening of open eye responses, we have investigated the contribution of GABA_B_ receptors in OD plasticity using cortical infusion of the antagonist SCH50911 and agonist baclofen in juvenile and adult cats. After the administration of the antagonist, we found that OD plasticity was impaired, while the agonist promoted OD plasticity, but only during the critical period. We performed these experiments in cats, instead of mice because cats are a better-developed model of both cortical infusion and OD plasticity [20–25], and they have much higher visual acuity and more prominent binocularity.

## Methods

### Animals and husbandry

Experiments were performed on 19 kittens (N = 3 Control, N = 3 PBS + MD 4–5 weeks, N = 5 SCH50911 + MD, N = 3 PBS + MD 7–8 weeks and N = 5 baclofen + MD; 4–8 weeks old; weight: 0.546 ± 0.037 kg) and 14 adult cats (N = 4 Adult Control, N = 3 Adult MD, N = 3 SCH50911 + MD and N = 4 baclofen + MD; > 1 year old; weight: 2.78 ± 0.226 kg) of both genders. This was the minimum sample size to ensure statistically valid results. All animals were naïve (not used for other experiments) and treatment groups were randomly assigned. All animals were obtained from institutional breeding colonies, and were individually housed in standard cages, except when: 1) pairs were placed together for breeding, or 2) kittens were housed with the dam. Animals had food and water *ad libitum* and were housed on a 12 hour/12 hour light-dark cycle. Good ventilation and sanitation were ensured in order to prevent post-operative infection. Cats were monitored daily for health status.

### Ethical statement

All experiments were carried out in accordance with the National Institutes of Health Guide for the Care and Use of Laboratory Animals, and were approved by the Institutional Animal Care and Use Committees of the University of Science and Technology of China or Yale University.

### Minipump implantation

All of the following operations can be seen in protocols.io (http://dx.doi.org/10.17504/protocols.io.h2db8a6). Animals were examined with an ophthalmoscope to ensure that they had no retinal disease or damage before each experiment. Details of our experimental methods have been described previously [20, 21]. Briefly, animals were given acepromazine (0.1 mg/kg, i.m.), atropine (0.04 mg/kg, i.m.) and dexamethasone (1 mg/kg, i.m.), then anesthetized with ketamine (25 mg/kg, i.m.) and xylazine (1.5 mg/kg, i.m.), intubated, mounted in a stereotaxic apparatus and prepared for surgery following standard aseptic techniques. Anesthesia was maintained with halothane or isoflurane (0.5–1.2%) delivered in a 2:1 mixture of nitrous oxide and oxygen. The animal’s core temperature was maintained at ~38°C using a homeostatically controlled heating pad. The electrocardiogram, blood oxygen saturation and end-tidal carbon dioxide were continuously monitored. A small craniotomy (~1.5 mm in diameter) was made over the left visual cortex 5 mm posterior to interaural zero and 2 mm lateral to midline. A hole was made in the dura and the tip of a 30 G cannula (Alzet brain infusion kit, Durect) was lowered into the cortex to a depth of 1.5–2 mm. The cannula was cemented in place and connected to an osmotic minipump (Alzet model 2001, Durect) containing one of the following solutions: vehicle alone (sterile 0.033 M phosphate-buffered saline, PBS), 20 mM baclofen in vehicle, or 20 mM SCH50911 in vehicle (drugs were obtained from Tocris Biosciences and Sigma). The minipump was inserted beneath the skin of the neck, the incision was sutured shut, and a topical lidocaine gel (2%) was applied to the incision site. After surgery, animals were given an antibiotic (Baytril, 2.5 mg/kg, i.m., once/day for three days) and an analgesic (Buprenex 0.01 mg/kg, i.m., twice/day for three days). Anesthesia and nitrous oxide were discontinued and the animal was allowed to recover on the heating pad. At the first sign of waking the animal was extubated, removed from the heating pad and observed until fully alert. The incision site was checked daily for any sign of opening or infection.

### Monocular deprivation

Early the next morning, about 12 hours after minipump implantation, the animal’s right eye was sutured shut with 4–0 silk under halothane or isoflurane (0.5–3%) anesthesia. A small bead of ophthalmic antibiotic ointment (Neosporin or chlortetracycline hydrochloride) was applied to the eye before closing. The deprived eye was checked daily for any sign of opening or infection.

### Electrophysiological recording

After four days of MD (4.5 days of drug infusion) in kittens or seven days of MD (7.5 days of drug infusion) in adults, animals were prepared for extracellular single unit recording. Surgical procedures incorporated the methods of Beaver et al. [21], Shou et al. [26], and/or An et al. [27]. Briefly, animals were given atropine (0.04 mg/kg, i.m.) and dexamethasone (1 mg/kg, i.m.), then anesthesia was induced with ketamine (25 mg/kg, i.m.), and a tracheotomy and venous catheterization were performed. All wounds and pressure points were treated with lidocaine (2%) and the animal was placed in a stereotaxic apparatus. Thereafter anesthesia was maintained with: pentobarbital (~3 mg/kg/h, i.v.), or halothane (0.5–3%, in a 2:1 mixture of nitrous oxide and oxygen) in kittens; or urethane (given as an initial dose of 30 mg/kg, i.v., followed by the infusion of ~20 mg/kg/h, i.v.) in adults. All animals were paralyzed with gallaminetriethiodide (8–10 mg/kg/h, i.v.) and placed on mechanical respiration. All intravenous solutions were delivered in a normal saline with glucose. Heart rate was monitored continuously as an indicator of anesthetic level. End-tidal carbon dioxide, blood oxygen saturation, and body temperature were monitored and maintained within normal physiological limits. The deprived eye was opened and 1–2 drops of phenylephrine (1%) and tropicamide (0.25%) were placed in each eye to retract the nictitating membrane and dilate the pupil. Both eyes were fitted with contact lenses to correct focus and prevent corneal desiccation.

The scalp was opened, the incision margins were treated with lidocaine (2%), and the minipump and cannula were removed and inspected to ensure that they were intact and functioning properly. A large craniotomy was made to expose the left primary visual cortex and the dura reflected to allow for six or more electrode penetrations. Epoxylite-insulated tungsten microelectrodes (3–5 MΩ, FHC Inc.) were advanced using a hydraulic micromanipulator (Narishige) angled at roughly 20° from vertical in an anterior to posterior direction to sample cells evenly across several OD columns.

The visual stimuli were moving sinusoidal gratings displayed on a CRT monitor (1024×768, 21 inch, 100 Hz, Sony) positioned 57 cm from the animal’s eyes, covering 40×30 degrees of visual angle. The luminance non-linearity of the monitor was corrected by an inverse-gamma function applied with software. The mean luminance of the monitor was ~60 cd/m^2^ and environmental luminance on the cornea was near 0.1 lux. The program used to generate the stimuli was coded in MATLAB (Mathworks).

For each neuron the preferred spatial frequency, direction and temporal frequency were determined. In kittens, using the optimal stimulus parameters, cells were assigned to ocular dominance categories according to the seven-category scheme of Hubel and Wiesel [28] based on the auditory discrimination of two independent listeners as described in previous studies [20, 21]. In adult cats, using the optimal stimulus parameters, the single unit activity evoked by the stimulation of each eye was recorded using an Igor program (S1 Fig). To avoid sampling bias, recorded single units were separated by at least 200 μm along the electrode track. Six or more penetrations were made in the drug-infused hemisphere (contralateral to the deprived eye). Using the method of Beaver [21], we compared drug treated neurons near the infusion site (1.0–3.5 mm distant) with unaffected neurons far from the infusion site (> 4.5 mm distant), providing an internal control for interanimal variability. After completing each penetration two or more electrolytic lesions were made along the electrode track to facilitate anatomical reconstruction. At the end of the recording experiment, the animal was transcardially perfused with normal saline, followed by 4% paraformaldehyde in normal saline. The visual cortex was sectioned at 50 μm intervals using a freezing microtome (Leica CM1950) and stained with cresyl violet. After the imaging of slices using a stereoscopic microscope (SZX-16, Olympus), penetrations were reconstructed to determine their distance from the infusion site and establish the laminar position of each recorded neuron (S1 Fig).

### Data analysis

Response quality was assessed using an activity index in which the level of visually driven and spontaneous activity were each rated on a three-point scale (1 = low to 3 = high). The OD index for each cell is calculated as (peak RE—spontaneous RE)/[(peak LE—spontaneous LE) + (peak RE—spontaneous RE)], where LE and RE represent the response from the left eye and the right eye respectively. Using the seven-category scheme of Hubel and Wiesel [28], a weighted ocular dominance (WOD) and binocularity index (BI) were then calculated as follows:

$$WOD\;=\;\left(1/6G_{2}+\;2/6G_{3}+\;3/6G_{4}+\;4/6G_{5}+\;5/6G_{6}+\;G_{7}\right)/N$$

$$BI=\left[1/3\left(G_{2}+\;G_{6}\right)\;+\;2/3\left(G_{3}+\;G_{5}\right)\;+\;G_{4}\right]/N$$

With *G*_(i)_ representing the number of cells in OD group i (i = 1–7; 1, deprived eye only and 7, non-deprived eye only) and *N* representing the total number of recorded cells [29]. Thus, a WOD score of 1 indicates all cells respond only to the non-deprived eye, while a WOD score of 0 means all cells respond only to the deprived eye; and a BI score of 0 means that all cells are monocularly-driven, while a BI score of 1 means that all cells are binocularly-driven. For all measures data are expressed as mean ± SEM.

Statistical analysis was performed with GraphPad Prism 5 and MATLAB. Differences between OD histograms were assessed using a χ^2^ test. Differences between two groups were assessed with *t*-test. Differences of WOD scores between near and far sites were evaluated with a paired *t*-test. Differences between three groups were evaluated with one-way ANOVA followed by Tukey post hoc test. Variation of WOD scores in different layers at near or far sites was evaluated with two-way ANOVA. Level of significance was p < 0.05.

## Results

### Blockade of GABA_B_ receptors impaired OD plasticity during the critical period

At the peak of the critical period for ocular dominance plasticity (4–5 weeks of age), kittens received an infusion of the GABA_B_ receptor antagonist SCH50911 (20 mM) into the left visual cortex (contralateral to the deprived eye). This concentration of SCH50911 is roughly three orders of magnitude higher than that required to block GABA_B_ receptors *in vitro* [30]. Previous studies [20–23] using compounds of similar molecular weight and solubility have shown that this concentration results in drug efficacy at near but not far sites. Control kittens received an infusion of vehicle only (0.033 M PBS).

**Fig 1:**
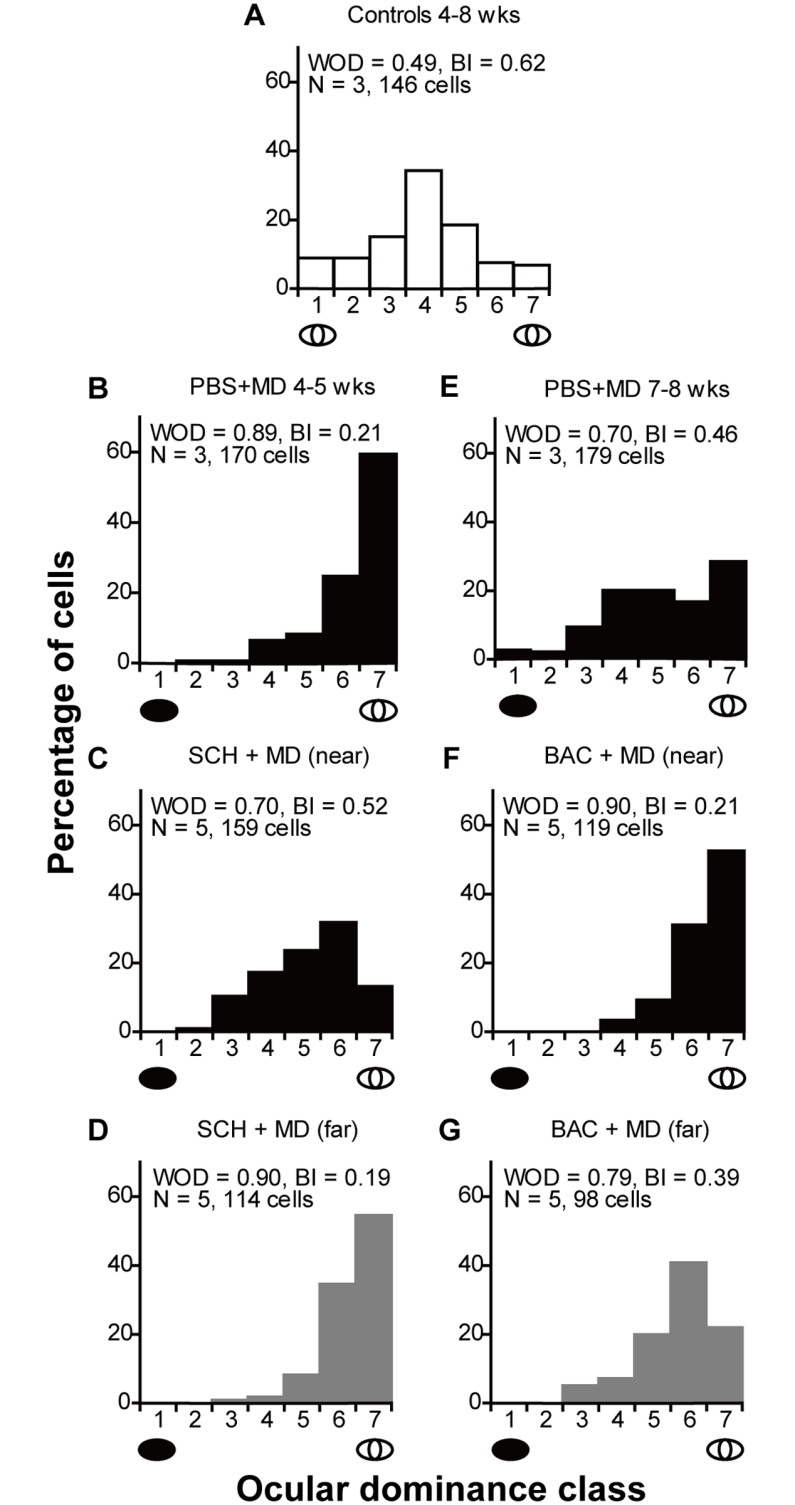
GABA_B_ receptor bidirectionally regulates OD plasticity during the critical period. **(A)** OD distribution for 4-8-week-old control kittens (N = 3). Note that the majority of cells receive input from both eyes. **(B-D)** OD distributions for 4-5-week-old kittens subject to 4 days of MD and infused with PBS, or the GABA_B_ receptor antagonist SCH50911 (SCH). Note that MD resulted in an OD shift toward the open eye (B) (p = 0, χ^2^ test), which was reduced near (C) but not far (D) from the SCH50911 infusion site (p = 1.14 × 10^−13^, χ^2^ test). **(E-G)** OD distributions for 7–8 weeks old kittens subject to 4 days of MD and infused with PBS, or the GABA_B_ receptor agonist baclofen (BAC). Note that MD at 7–8 weeks of age (E) induced a weaker OD shift than in younger animals (p = 1.56 × 10^−11^, χ^2^ test, cf. 1B and 1E). However, this OD shift was enhanced near (F) but not far (G) from the baclofen infusion site (p = 2.66 × 10^−5^, χ^2^ test). WOD = weighted ocular dominance. BI = binocularity index. N = number of animals.

Consistent with previous studies [31], cortical cells were driven by both eyes in control kittens without MD (WOD = 0.49, BI = 0.62; Fig 1A). Four days of MD in PBS treated animals, induced a significant OD shift toward the non-deprived eye (WOD = 0.89, BI = 0.21; p = 0.0, χ^2^ test; Fig 1A and 1B) (data from near and far sites in PBS + MD controls were not significantly different, so we have combined data from all penetrations in this group). In contrast, MD combined with SCH50911 treatment significantly reduced the OD shift at near sites (WOD = 0.70, BI = 0.52; Fig 1C) relative to that at far sites (WOD = 0.90, BI = 0.19; p = 1.14 × 10^−13^, χ^2^ test; Fig 1D), indicating that the blockade of GABA_B_ receptors impairs OD plasticity during the critical period. Moreover, the OD distribution of cells at far sites in SCH50911 + MD treated kittens (Fig 1D) was similar to that in PBS + MD treated kittens (p = 0.2696, χ^2^ test; Fig 1B), confirming that the drug had no significant impact on OD at far sites.

### Activation of GABA_B_ receptors promoted OD plasticity late in the critical period

To investigate whether the residual plasticity beyond the peak of the critical period could be enhanced by the activation of GABA_B_ receptors, we treated kittens at 7–8 weeks of age with either 20 mM baclofen or PBS vehicle. As expected, MD in PBS treated kittens induced a significant OD shift toward the non-deprived eye (WOD = 0.70, BI = 0.46; p = 2.94 × 10^−8^, χ^2^ test; Fig 1E), although the OD shift was less pronounced than that observed in 4–5 week-old kittens (p = 1.56 × 10^−11^, χ^2^ test; cf Fig 1B and 1E). Baclofen infusion significantly increased this OD shift at near sites (WOD = 0.90, BI = 0.21; Fig 1F) verses far sites (WOD = 0.79, BI = 0.39; p = 2.66 × 10^−5^, χ^2^ test; Fig 1G). These findings suggest that the activation of GABA_B_ receptors enhanced the residual OD plasticity present late in the critical period.

**Fig 2:**
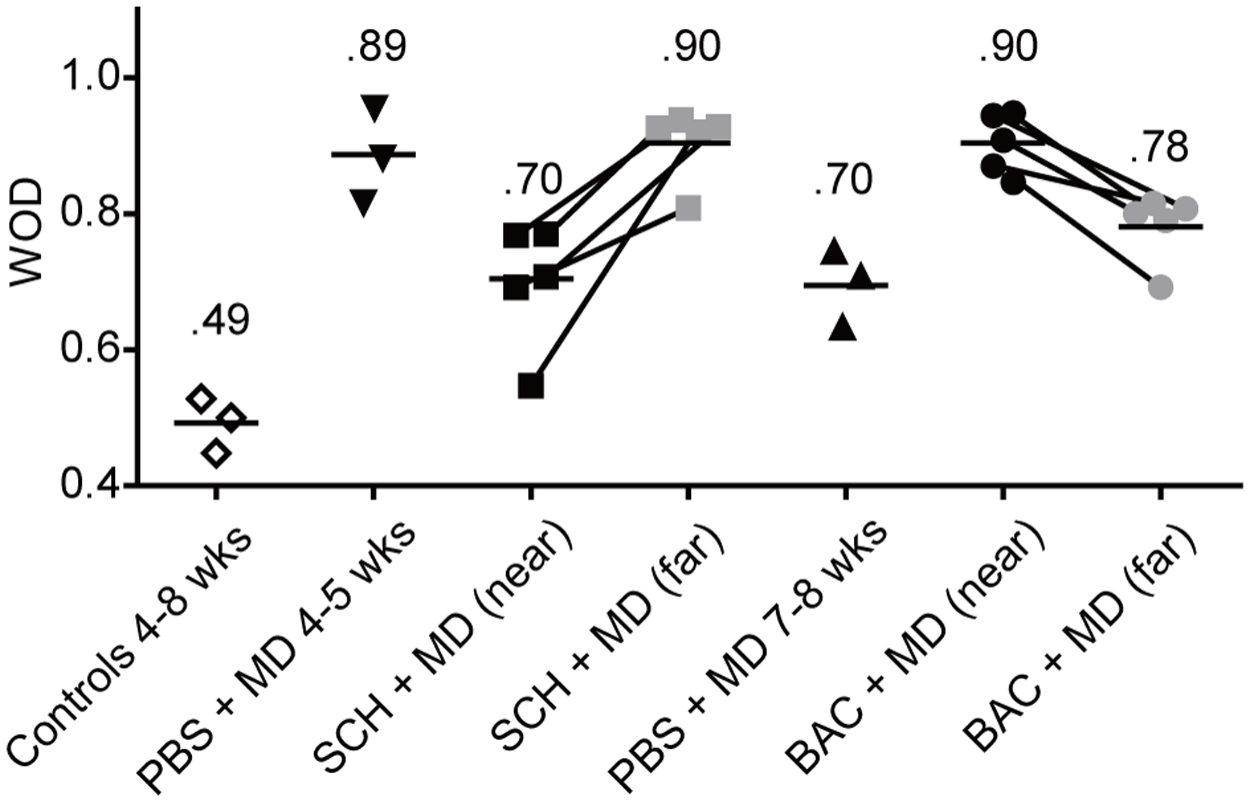
WOD scores for each kitten in each treatment group. Discrete symbols represent WOD scores obtained for individual animals, while the labeled bar indicates the group mean. Note that WOD scores were significantly lower near verses far from the SCH50911 infusion site (p = 0.0098, paired *t*-test). In contrast, WOD scores near the baclofen infusion site were significantly higher than those far from the baclofen infusion site (p = 0.003, paired *t*-test). Conventions as in Fig 1.

Fig 2 shows a summary of WOD scores for each kitten. WOD scores at far sites in SCH50911 + MD and baclofen + MD treated animals were not significantly different from those in age-matched PBS + MD controls (0.90 ± 0.02 vs 0.89 ± 0.04 and 0.78 ± 0.02 vs 0.70 ± 0.03; p = 0.70 and p = 0.07, respectively; *t*-test), confirming that neurons at the far sites were not influenced by the drug and could be used as internal controls to exclude inter-animal variability. After SCH50911 + MD treatment, WOD scores at near sites were significantly lower than those at far sites (0.70 ± 0.04 vs 0.90 ± 0.02; p = 0.0098, paired *t*-test). In contrast, in baclofen + MD treated kittens, WOD scores at near sites were significantly higher than those at far sites (0.90 ± 0.02 vs 0.78 ± 0.02; p = 0.003, paired *t*-test). Taken together, these findings provide evidence for an endogenous GABA_B_ receptor-dependent mechanism in the regulation of OD plasticity during the critical period.

Infusion of a GABA_B_ receptor agonist or antagonist may also alter other neuronal response properties. However, an analysis of receptive field area showed no significant difference between control and drug-treated groups (Control vs SCH50911, p = 0.95; Control vs baclofen, p = 0.06, *t*-tests; S2 Fig). Additionally, the vigor of visually driven responses (Control vs SCH50911, p = 0.06; Control vs baclofen, p = 0.72, *t*-tests) and level of spontaneous activity (Control vs SCH50911, p = 0.50; Control vs baclofen, p = 0.35, *t*-test; S2 Fig) were unchanged. Therefore, any differences in cortical plasticity are not likely to result from the disruption of the receptive field properties or neuronal responsiveness after drug treatment.

### Manipulation of GABA_B_ receptor activity failed to promote OD plasticity in adult cats

Several studies have shown that reducing cortical GABAergic inhibition promotes OD plasticity in adults [4–7]. To investigate whether the inhibition mediated by GABA_B_ receptors promotes adult OD plasticity, we infused SCH50911 or baclofen into the primary visual cortex of MD adult cats (>1 year of age). We used the same concentrations of SCH50911 (20 mM) and baclofen (20 mM) for adult cats that we had for kittens, however, because adult OD plasticity is reportedly less robust than that observed during the critical period [32–34], we increased the period of MD to 7 days (the limit of minipump function for this concentration of drugs). Additionally, we used urethane anesthesia which has previously enabled the detection of adult plasticity in the visual cortex even after brief periods of deprivation in mice [33, 35].

**Fig 3:**
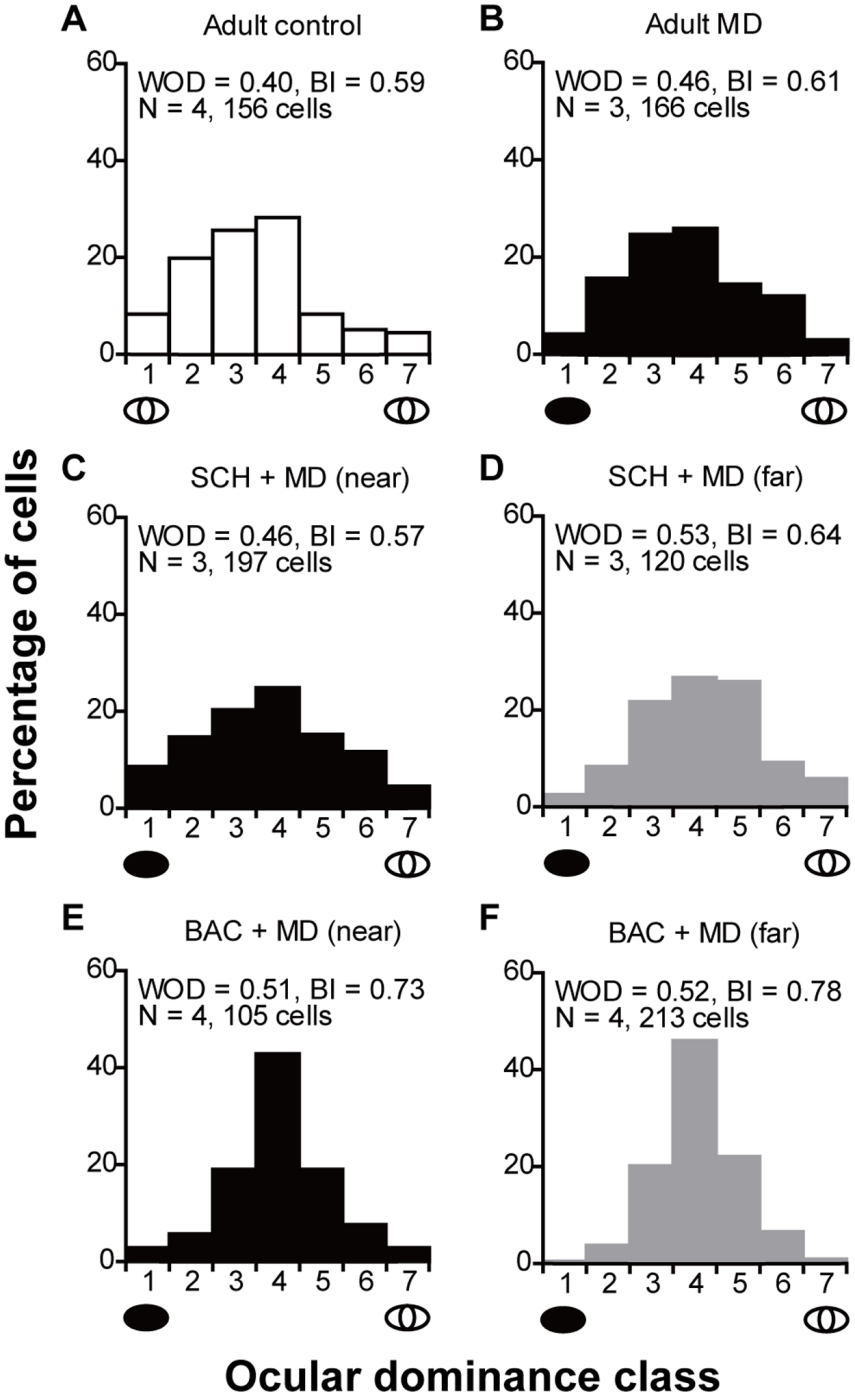
Manipulation of GABA_B_ receptor activity failed to induce OD plasticity in adult cats. **(A-B)** OD distributions for intact control adults (A) and adults with 7 days of MD (B) were similar (p = 0.0978, χ^2^ test), indicating that MD alone failed to induce adult OD plasticity. **(C-D)** OD distributions for adult cats with 7 days of MD for sites near (C) and far (D) from SCH50911 infusion were similar (p = 0.0596, χ^2^ test), and the majority of cells received input from both eyes at near and far sites, which was similar to control adults and adults with 7 days of MD (cf. 3A, 3B, 3C and 3D). **(E-F)** OD distributions for adult cats with 7 days of MD for sites near (E) and far (F) from baclofen infusion were also similar (p = 0.4162, χ^2^ test). Note again that the majority of cells received input from both eyes at sites near and far from baclofen infusion, which was similar to adult controls and adults with 7 days of MD (cf. 3A, 3B, 3E and 3F). Conventions as in Fig 1.

**Fig 4:**
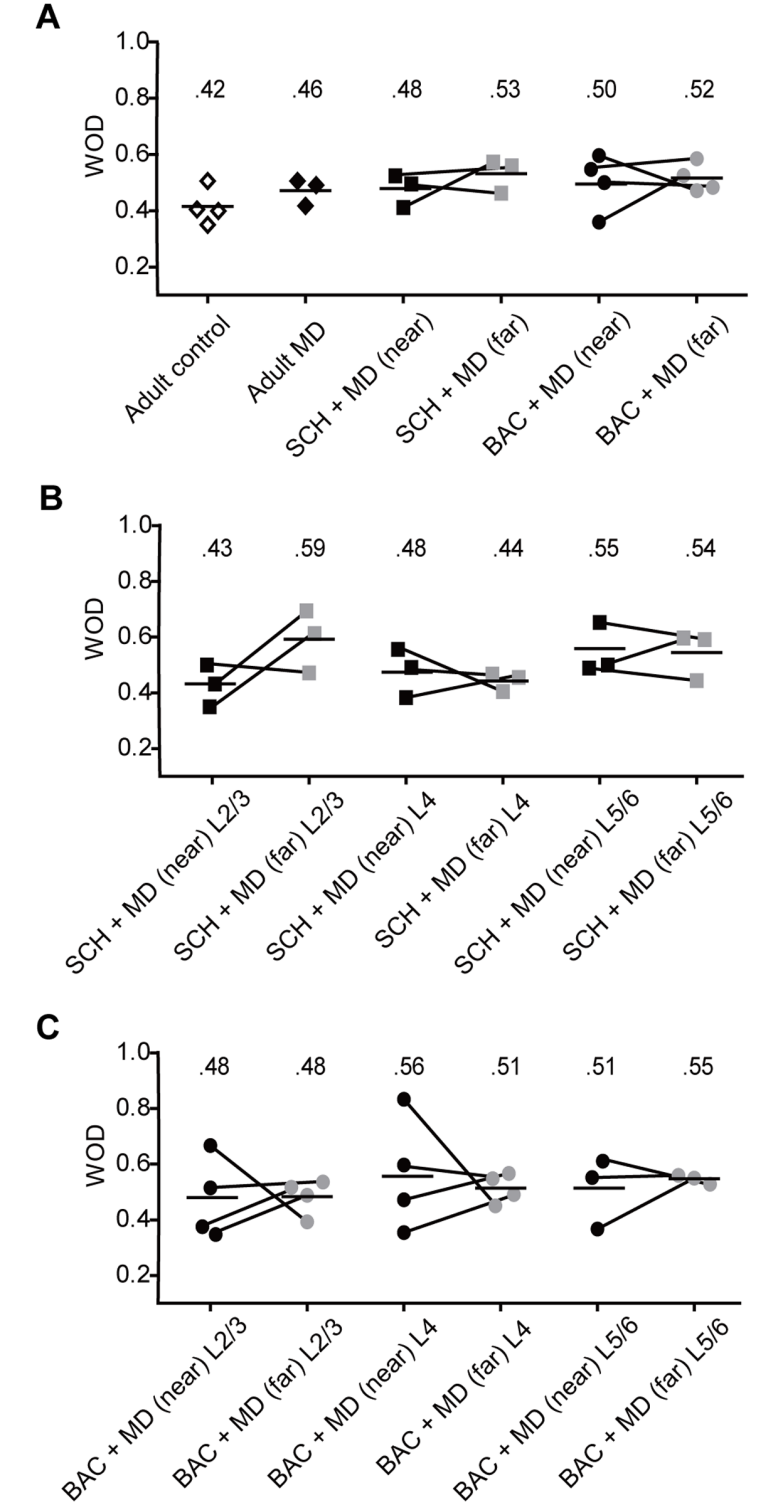
WOD scores for all cats and for each treatment in each layer. **(A)** WOD scores for each adult in each treatment group for all layers. Discrete symbols represent WOD scores obtained for individual animals, while the labeled bar indicates the group mean. WOD scores of adult controls were not significantly different from adult MD (p = 0.41, *t*-test). And the scores did not differ between MD and treatment groups (p = 0.81, one-way ANOVA). WOD scores at near sites did not differ from those at far sites in SCH50911 + MD or baclofen + MD treatment groups (p = 0.44 and 0.82, paired *t*-test), indicating that blockade or activation of GABA_B_ receptors in conjunction with MD failed to induce adult OD plasticity. **(B-C)** WOD scores for each adult in each treatment group as a function of cortical layer for SCH50911 + MD (B) and Baclofen + MD (C) treated cats. Note that the values for each layer at near and far sites were not different from each other for the SCH50911 + MD treatment group (p = 0.13, two-way ANOVA), or the Baclofen + MD treatment group (p = 0.80, two-way ANOVA). L = layer. Conventions as in Fig 1.

Consistent with previous studies in adult untreated control cats [31, 36], most cells were driven by both eyes (WOD = 0.40, BI = 0.59; Fig 3A), and this was not altered by 7 days of MD (WOD = 0.46, BI = 0.61; p = 0.0978, χ^2^ test; Fig 3B). Neither SCH50911 (Fig 3C and 3D) which may abolish the GABA_B_ receptor-mediated inhibitory currents at postsynaptic sites, nor baclofen (Fig 3E and 3F) which may decrease GABA release at pre-synaptic sites, impacted OD after 7 days of MD in adults (Fig 3C and 3D: p = 0.0596, χ^2^ test; Fig 3E and 3F: p = 0.4162, χ^2^ test). The WOD scores of adult controls were not significantly different from those of adult MD animals (0.42 ± 0.03 vs 0.46 ± 0.04; p = 0.41, *t*-test; Fig 4A). The WOD scores for cells at near sites did not significantly differ from those at far sites for either SCH50911 or baclofen-treated groups (0.48 ± 0.03 vs 0.53 ± 0.04 and 0.50 ± 0.05 vs 0.52 ± 0.03; p = 0.44 and p = 0.82, respectively, paired *t*-test; Fig 4A). Furthermore, a one way ANOVA did not indicate any significant difference between drug conditions (F_2, 7_ = 0.22, p = 0.81; Fig 4A).

Previous studies, however, have shown that OD plasticity differs by layer [13, 37–39], and is especially prominent in extragranular layers [37]. Thus, we analyzed WOD by layer for each drug treatment in adults. In SCH50911 + MD treated cats (Fig 4B), WOD scores: for L2/3 were 0.43 ± 0.04 (near) vs. 0.59 ± 0.07 (far), for L4 were 0.48 ± 0.05 (near) vs. 0.44 ± 0.02 (far), and for L5/6 were 0.55 ± 0.05 (near) vs. 0.54 ± 0.05 (far). A two-way ANOVA of this data with layer (L2/3 vs L4 vs L5/6) and site (near vs far) as within-subjects factors did not reveal significant effects of layer (F_2, 12_ = 1.57, p = 0.25), site (F_1, 12_ = 1.14, p = 0.31), or any significant interaction (F_2, 12_ = 2.43, p = 0.13). Similarly in baclofen + MD treated animals (Fig 4C), WOD scores: for L2/3 were 0.48 ± 0.07 (near) vs. 0.48 ± 0.03 (far), for L4 were 0.56 ± 0.10 (near) vs. 0.51 ± 0.03 (far), and for L5/6 were 0.51 ± 0.07 (near) vs. 0.55 ± 0.01 (far). A two-way ANOVA of this data did not show a significant effect of layer (F_2, 16_ = 0.51, p = 0.61), site (F_1, 16_ = 0, p = 0.97), or any significant interaction (F_2, 16_ = 0.23, p = 0.80). Together, these results suggest that 7 days of MD, even in combination with the alteration of GABA_B_ receptor-mediated inhibition, is insufficient to induce OD plasticity in adult cats.

## Discussion

GABAergic inhibition is an important mechanism underlying cortical plasticity. The role of GABA_A_, but not GABA_B_, receptors has been rigorously examined. Our results show that GABA_B_ receptor blockade reduces, while GABA_B_ receptor activation enhances, the OD shift induced by MD in kittens. These findings indicate that OD plasticity can be bidirectionally regulated by an endogenous GABA_B_ receptor-dependent mechanism. Furthermore, we find that this mechanism regulates juvenile but not adult OD plasticity. Thus, our results add to the growing body of evidence suggesting that GABA_B_ receptors contribute specifically to critical period OD plasticity in the visual cortex.

GABA_B_ receptor activation may promote OD plasticity via the enhancement of excitatory LTD and/or blockade of inhibitory LTP. Several mechanisms have been proposed to contribute to the weakening of deprived eye connections following MD during the critical period, including LTD of excitatory, or LTP of inhibitory, inputs onto pyramidal cells [2, 40, 41]. MD *in vivo* mimics the properties of excitatory LTD *in vitro* [42]. Both excitatory LTD and OD plasticity require activation of the cAMP/PKA/CREB pathway [21, 43–46]. Significantly, GABA_B_ receptor agonists potentiate cAMP activation [47]. Moreover, inhibitory LTP at fast-spiking cell to pyramidal cell synapses is mediated by a postsynaptic GABA_B_ receptor-dependent process [11, 12], which is expressed during the critical period and is occluded by MD [12, 48]. Additionally, GABA_B_ receptors have been shown to inhibit L-type calcium channels [49], and facilitate metabotropic glutamate receptor-mediated excitatory LTD [50]. The inhibition of L-type calcium channels impaired the induction of inhibitory LTP, and consequently alters the capacity for plasticity in a pyramidal cell at convergent excitatory synapses—shifting their expression from LTP to LTD [12]. Finally, postsynaptic GABA_B_ receptors can activate Kir3 potassium channels which hyperpolarize the membrane and shunt excitatory currents [8]. Thus, activation of GABA_B_ receptors may orchestrate the suppression of pyramidal cell excitability through the coordinated postsynaptic modulation of inhibitory and excitatory transmission. Taken together, these findings suggest that GABA_B_ receptor-dependent mechanism may engage the expression of OD plasticity *in vivo*.

GABA_B_ receptors may also modulate OD plasticity indirectly through cannabinoid receptors. GABA_B_ receptors are co-localized with cannabinoid receptors in presynaptic terminals, and show significantly reduced activity in cannabinoid receptor knockout mice [51]. Previous work has shown that cannabinoid receptor blockade impairs OD plasticity by preventing the depression of deprived eye response [13]. In the visual cortex, the expression of presynaptic cannabinoid receptor-dependent inhibitory LTD and the expression of OD plasticity are correlated. Both are robust during the critical period, and show a rapid decline as the critical period comes to a close [52, 53]. Finally, GABA_B_ receptors are known to mediate presynaptic cannabinoid receptor-dependent inhibitory LTD [15]. Hence, our results may reflect an alteration of pyramidal cell excitability via presynaptic regulation of inhibitory transmission and postsynaptic regulation of excitatory transmission.

It is generally accepted that regulating inhibitory output through GABA_A_ receptors can affect the timing of the critical period. Notably, pharmacological enhancement of inhibition with diazepam (an agonist of GABA_A_ receptors) prevents OD plasticity during the critical period [54]. This contrasts with our results showing that the GABA_B_ receptor agonist baclofen enhances OD plasticity. Although GABA_B_ receptors are localized at both pre- and post-synaptic sites, our results suggest the effect of GABA_B_ receptors may be primarily mediated through a pre-synaptic mechanism. If this was correct, following the activation of pre-synaptic GABA_B_ receptors by baclofen, GABA release would be decreased which would in turn decrease the activity of GABA_A_ receptors. This could explain the contrasting effects of baclofen and diazepam. A final possibility involves observations that GABA_B_ receptors are located on the presynaptic terminals of thalamocortical axons [55] while GABA_A_ receptors are not [56]. This would suggest that input from thalamocortical afferents could play a key role in the regulation of binocular vision during critical period development [57]. Strong staining for GABA_B_ R2 subunits in the thalamocortical axon tracts only presents at an early developmental stage and not in adults [58]. Thus, a GABA_B_ dependent presynaptic mechanism might directly change the visual input from dorsal lateral geniculate nucleus to visual cortex, and thereby influence the expression of OD plasticity during the critical period but not in adults.

The fact that we did not observe OD plasticity in adult cats with either MD alone or in combination with the manipulation of GABA_B_ receptor activity may result from the alteration of the subunit composition and functional properties of cortical GABA_B_ receptor during development. Between birth and adulthood the expression of the R1a isoform of GABA_B_ receptor decreases by a factor of five, while that of the R1b isoform doubles [59, 60]. Given that the R1a isoform is predominant at presynaptic site, and the R1b isoform is mainly postsynaptic [19, 61], this developmental shift in subunit expression suggests the presynaptically mediated GABA_B_ receptor-dependent mechanisms would be down regulated in adults. Moreover, adult OD plasticity appears more robust in rodents than cats. Induction of OD plasticity requires just five days of MD in adult rodents [32, 62], but three months of MD in adult cats [37]. The magnitude of adult OD plasticity remains constant in rodents [33], but diminishes with age in cats [37]. Furthermore, the suppression of intracortical inhibition in adult rodents not only promotes OD plasticity, but also reduces the expression of chondroitin sulfate proteoglycans [7], a component of extracellular matrix thought to restrict adult OD plasticity [63]. However, in cats, the degradation of chondroitin sulfate proteoglycans induced by MD is insufficient to promote recovery of function in the deprived eye [64]. Collectively, these factors may explain why the GABA_B_ receptor-dependent mechanisms we observed contributing to critical period plasticity are subsequently lost in adults.

## Conclusions

While the precise role of GABA_B_ receptors in OD plasticity remains to be elucidated, this discovery provides a further understanding of critical period mechanisms. Baclofen is the only available medication targeting GABA_B_ transmission and is used as a therapeutic treatment for anxiety, depression, epilepsy and cognitive disorders [65–67]. In this study, we found that baclofen enhanced OD plasticity during the critical period, and this could broaden its spectrum of therapeutic applications.

## Supporting information

S1 Checklist Completed “The ARRIVE Guidelines Checklist” for reporting animal data in this manuscript. (PDF)

S1 Fig Raw traces of single unit recordings and histological reconstruction of electrode tracks. **(A)** Examples of responses evoked by 10 trails of moving sinusoidal gratings. Scale bars are 100 μV, 0.5 s. **(B)** Microscope image of a histologically processed sagittal section showing the location of minipump (ellipse), near site (red arrows) and far site (black arrows). Scale bar = 1 mm.
(TIF)

S2 Fig Visual response properties of single units in drug-infused animals were not different from controls. **(A)** Receptive field (RF) area is similar in neurons without drug (Control), and after SCH50911 (SCH) or baclofen (BAC) infusion. The histogram is based on 205 cells (Control), 156 cells (SCH), and 133 cells (BAC). **(B)** The vigor of visually driven (left column) and spontaneous activity (right column) are also similar in these groups, as rated using a three-point activity index (1 = low to 3 = high; see Methods). The histogram is based on 176 cells (Control), 156 cells (SCH), 120 cells (BAC).
(TIF)
