# Supplementary figures and images for: GABAB receptor-dependent bidirectional regulation of critical period ocular dominance plasticity in cats
Source: PLoS One. 2017 Jun 29;12(6):e0180162. doi: 10.1371/journal.pone.0180162 (PMC5491141; doi:10.1371/journal.pone.0180162)

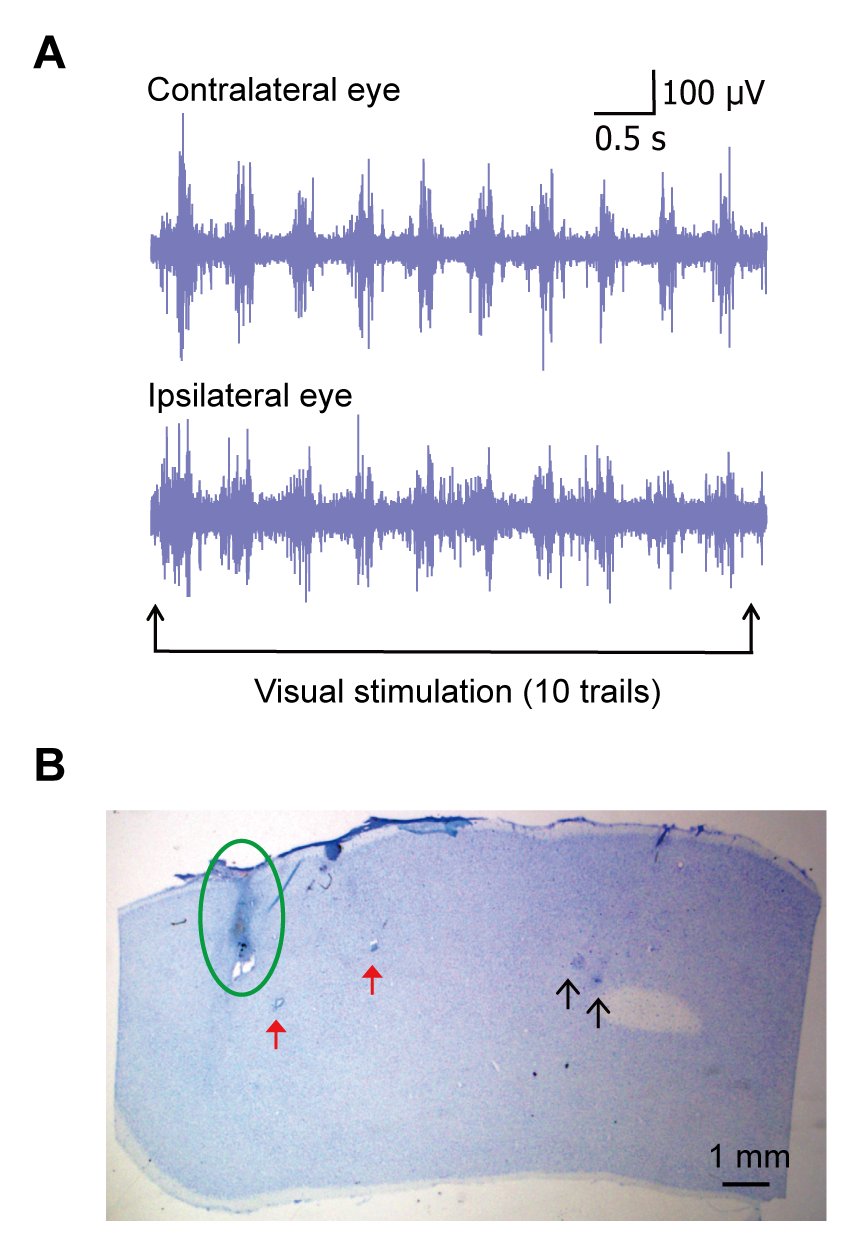

Supplement: S1 Fig — (A) Examples of responses evoked by 10 trails of moving sinusoidal gratings. Scale bars are 100 μV, 0.5 s. (B) Microscope image of a histologically processed sagittal section showing the location of minipump (ellipse), near site (red arrows) and far site (black arrows). Scale bar = 1 mm. (TIF) [file pone.0180162.s002.tif]

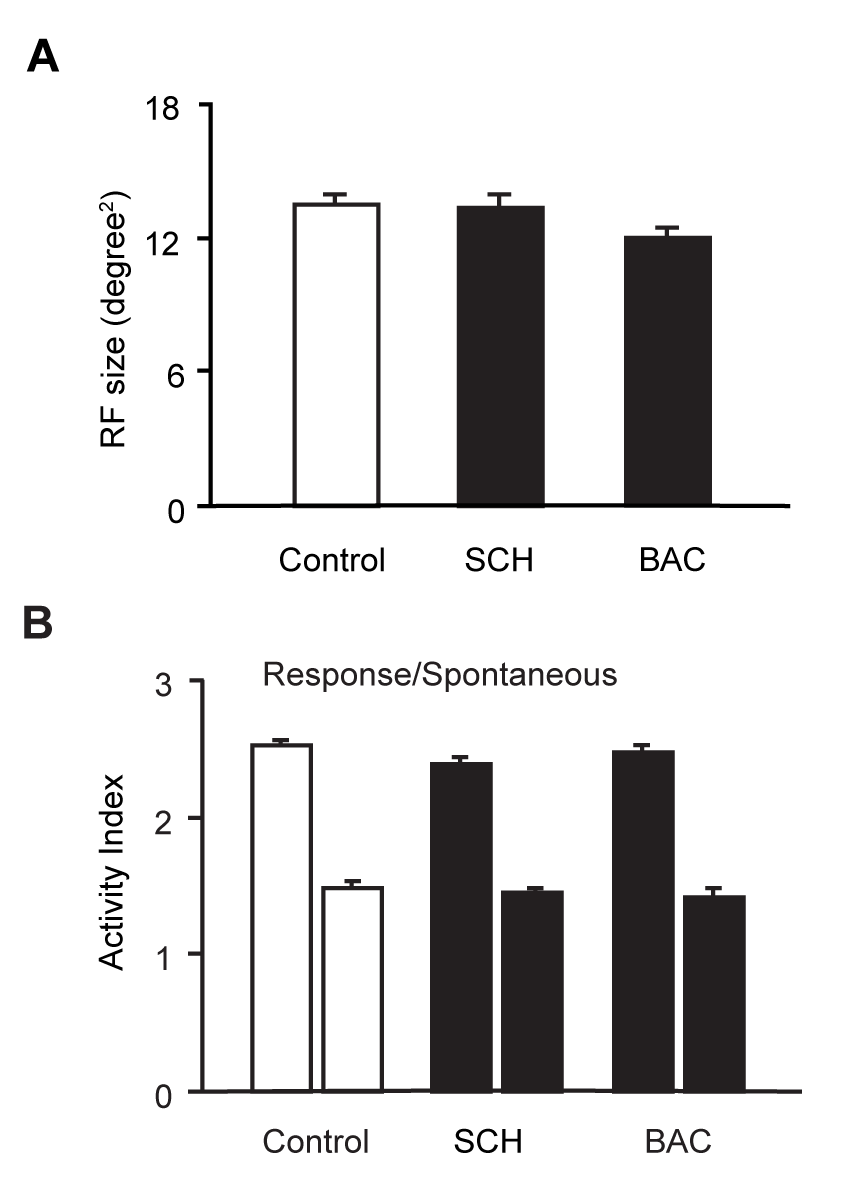

Supplement: S2 Fig — (A) Receptive field (RF) area is similar in neurons without drug (Control), and after SCH50911 (SCH) or baclofen (BAC) infusion. The histogram is based on 205 cells (Control), 156 cells (SCH), and 133 cells (BAC). (B) The vigor of visually driven (left column) and spontaneous activity (right column) are also similar in these groups, as rated using a three-point activity index (1 = low to 3 = high; see Methods). The histogram is based on 176 cells (Control), 156 cells (SCH), 120 cells (BAC). (TIF) [file pone.0180162.s003.tif]
